# The Potential of Automatic Word Comparison for Historical Linguistics

**DOI:** 10.1371/journal.pone.0170046

**Published:** 2017-01-27

**Authors:** Johann-Mattis List, Simon J. Greenhill, Russell D. Gray

**Affiliations:** 1 Centre des Recherches Linguistiques sur l’Asie Orientale, École des Hautes Études en Sciences Sociales, 2 Rue de Lille, 75007 Paris, France; 2 Department for Linguistic and Cultural Evolution, Max Planck Institute for the Science of Human History, Kahlaische Straße 10, 07743, Jena, Germany; 3 ARC Centre of Excellence for the Dynamics of Language, Australian National University, Canberra, 2600, Australia; Massachusetts Institute of Technology, UNITED STATES

## Abstract

The amount of data from languages spoken all over the world is rapidly increasing. Traditional manual methods in historical linguistics need to face the challenges brought by this influx of data. Automatic approaches to word comparison could provide invaluable help to pre-analyze data which can be later enhanced by experts. In this way, computational approaches can take care of the repetitive and schematic tasks leaving experts to concentrate on answering interesting questions. Here we test the potential of automatic methods to detect etymologically related words (cognates) in cross-linguistic data. Using a newly compiled database of expert cognate judgments across five different language families, we compare how well different automatic approaches distinguish related from unrelated words. Our results show that automatic methods can identify cognates with a very high degree of accuracy, reaching 89% for the best-performing method *Infomap*. We identify the specific strengths and weaknesses of these different methods and point to major challenges for future approaches. Current automatic approaches for cognate detection—although not perfect—could become an important component of future research in historical linguistics.

## Introduction

Historical linguistics is currently facing a dramatic increase in digitally available datasets [1–5]. The availability of data for more and more languages and language families challenges the ways in which we traditionally compare them. The comparative method has been the core method for linguistic reconstruction for the past 200 years [6], and is based on manually identifying systematic phonetic correspondences between many words in pairs of languages. However, there are too few expert historical linguists to analyse the world’s more than 7500 languages [7] and, consequently, only a small percentage of these languages have been thoroughly investigated leaving us in the dark about their history and relationships. This becomes especially evident in largely understudied linguistic areas like New Guinea, parts of South America, or the Himalayan region, and our lack of knowledge about these languages has immediate implications for our understanding of human prehistory.

Over the last two decades computational methods have been become more prevalent in historical linguistics. Advocates of computational methods emphasize the speed and replicability as the main advantage of computational techniques [8, 9]. However, sceptics criticise the validity and accuracy of these methods as lagging far behind those achieved by human experts. [10]. One approach in computational historical linguistics is to design fully-automated methods to identify language relationships with no input from researchers [11, 12]. Although these methods may provide interesting insights into linguistic macroareas [13], their “black-box” character makes it difficult to evaluate the results, as judgements about sound correspondences and decisions of cognacy are hidden. This opacity makes it difficult to improve the algorithms. More problematically, however, it limits the scientific value of these methods, as we do not just want to know how languages are related, but why and which pieces of evidence support this conclusion. As a result, there is much suspicion about these methods in historical linguistics [14–16].

Another approach—the one we take here—is to opt for a computer-assisted framework. In contrast to fully automated frameworks, computer-assisted frameworks seek to support and facilitate the task of language comparison by using human expertise where available to correct errors and improve the quality of the results. One of the core tasks of the comparative method is the identification of cognate words in multiple languages. If two words are cognate, this means that they are genetically related, and have descended from a common ancestor [17]. Cognate identification, along with the identification of regular sound correspondences, is the basis for proving that two or more languages are genetically related. It is also the basis for the reconstruction of ancestral word forms in historically unattested languages, and for the genetic classification of language families. In practice, cognate identification is a time-consuming process that is based on an iterative manual procedure where cognate sets are proposed, evaluated, and either kept or rejected [18].

This process of manual cognate identification should be an ideal candidate for computer-assisted tasks. As a possible workflow, scholars could first run an automatic cognate detection analysis and then edit the algorithmic findings. Even an iterative workflow in which the data is passed between computers and experts would be fruitful. An important question which arises in this context concerns the quality of automatic methods for cognate detection: Are these methods really good enough to provide concrete help to a highly trained expert? In order to find an answer to this question, we tested four publicly available methods and one newly proposed method for automatic cognate detection on six test sets covering five different language families, evaluated the performance of these methods, and determined their shortcomings.

## Materials and Methods

### Materials

There are few datasets available for testing the potential of cognate detection methods on language data, As such, testing algorithms run the risk of *over-fitting*. When developing an algorithm, one usually *trains* it on some datasets. If those datasets are afterwards used to also test the algorithm, the accuracy should be quite high, but we cannot tell whether the method will work on datasets apart from the ones on which the algorithm was trained. For this reason, it is important to split the available data into a training set and a test set. In our case, the training set will be used to determine the best parameters for each of the algorithms we test, while the test set will be used to carry out the actual test of cognate recovery.

For this study, we took training data from existing sources [19], while a new test dataset was compiled from scratch. The new test set consists of six datasets from five language families. These data were collected from different sources, including published datasets [3, 20–23], books [24], and ongoing research by scholars who allowed us to use parts of their data in advance (Uralex project, [25]). All datasets were formatted to tabular format and semi-automatically cleaned for various kinds of errors, like misspelled phonetic transcriptions, empty word slots, or obviously erroneous cognate judgments. We further linked all languages to Glottolog [7], and all wordlist concepts to the Concepticon [26].

Table 1 lists all datasets along with additional details, such as the number of words, concepts, languages, and cognate sets in the data. The diversity index given in the last column of the table is calculated by dividing the difference between cognate sets and meanings with the difference between words and meanings [19]. This score, which ranges between 0 and 1, indicates whether large numbers of words in a given dataset are unrelated (high index) or are cognate (low index). As can be seen from the diversity indices listed in the table, our test sets have varying degrees of diversity, ranging from 0.07 (Romance, Saenko, 2015) to 0.57 (Uralic).

**Table 1:** Test data used in our study.

Dataset	Words	Conc.	Lang.	Cog.	Div.
Bahnaric (Sidwell, 2015) [20]	4546	200	24	1055	0.20
Chinese (Běijīng Dàxué, 1964) [24]	3653	180	18	1231	0.30
Huon (McElhanon, 1967) [22]	1668	139	14	855	0.47
Romance (Saenko, 2015) [21]	4853	110	43	465	0.07
Tujia (Starostin, 2013) [23]	513	109	5	179	0.17
Uralic (Syrjänen et al, 2013) [25]	1401	173	7	870	0.57
TOTAL	16634	911	111	4655	0.30

As mentioned above, training data is needed for parameter estimation. The key parameter we need to estimate is the *best thresholds* for cognate identification in some of the methods. As training data we employed the collection of benchmark datasets for automatic cognate detection by List [19], which also covers six datasets from five language families. Details for this dataset (number of words, concepts, languages, cognate sets, and the diversity index) are given in Table 2. This dataset is available online at http://dx.doi.org/10.5281/zenodo.11877.

**Table 2:** Training data used in our study.

Dataset	Words	Conc.	Lang.	Cog.	Div.
Austronesian (Greenhill et al., 2008) [1]	4358	210	20	2864	0.64
Bai (Wang, 2006) [27]	1028	110	9	285	0.19
Chinese (Hóu, 2004) [28]	2789	140	15	1189	0.40
IndoEuropean (Dunn, 2012) [2]	4393	207	20	1777	0.38
Japanese (Hattori, 1973) [29]	1986	200	10	460	0.15
ObUgrian (Zhivlov, 2011) [30]	2055	110	21	242	0.07
TOTAL	16609	977	95	6817	0.30

### Methods

#### Automatic Cognate Detection

Many methods for automatic cognate detection have been proposed in the past (see Table 3 below). Unfortunately, only a few of these methods qualify as candidate methods for computer-assisted language comparison, since the majority are either (a) not able to analyse multiple languages at once, (b) have further requirements making their use more complicated [31, 32] e.g. require a user-specified reference phylogeny (and therefore assume that language groupings are already known), or need extensive training sets, or (c) are not freely available (see Table 3).

**Table 3:** Recent approaches to cognate detection. A plus “+” indicates that the algorithm meets the requirement, a minus “-” indicates that its failure. ML (multilingual) refers to the ability of an algorithm to identify cognate words across more than two languages at the same time. RQ (requirements) refers to additional requirements apart from the raw word list data, such as needing reference phylogenies or extensive training data. FA (free availability) means that the method has a useable public implementation.

Cognate Detection Approach	ML?	RQ?	FA?
Mackay and Kondrak, 2005, [34]	-	+	-
Bergsma and Kondrak, 2007, [35]	+	+	-
Turchin et al., 2010, [44]	+	+	+
Berg-Kirkpatrick and Klein, 2011, [36]	-	+	-
Hauer and Kondrak, 2011, [37]	+	+	-
Steiner et al., 2011, [38]	+	+	-
List, 2014, [19]	+	+	+
Beinborn et al., 2013, [31]	-	-	-
Bouchard-Côté, et al. 2013, [32]	+	-	-
Rama, 2013, [39]	-	+	-
Ciobanu and Dinu, 2014, [40]	-	+	-
Jäger and Sofroniev 2016, [41]	+	-	-

We decided to take four publicly available methods as the basis of our test study, the Turchin Method, the Edit Distance Method, the SCA Method, and the LexStat Method. Additionally, we tested a modified version of the LexStat method which we call Infomap. In this modified version of LexStat we introduced an improved partitioning method based on the Infomap algorithm for community detection [33]. All methods are presented in more detail below.

The four publicly available methods are all implemented as part of the same software package (LingPy, http://lingpy.org, [42]), and represent different degrees of algorithmic sophistication and closeness to linguistic theory, with the Turchin Method being very simple and computationally extremely fast, and the LexStat Method being rather complex and time-consuming. For the usage of the fifth method, we wrote a small LingPy plugin which builds on the python-igraph package (http://igraph.org/python-igraph/, [43], see details below) and is provided along with our supplementary material.

#### Cognate Detection following Turchin et al [44]

The Turchin method (also called *Consonant Class Matching* approach) was proposed by Turchin et al. [44]. In this method, the consonants of the words are converted to one of 10 possible consonant classes. The idea of consonant classes (also called sound classes) was proposed by Dolgopolsky [45], who stated that certain sounds occur more frequently in correspondence relation than others and could therefore be clustered into classes of high historical similarity. In the approach by Turchin et al., two words are judged to be cognate, if they match in their first two consonant classes.

#### Cognate Detection using the Edit Distance approach

**Fig 1:**
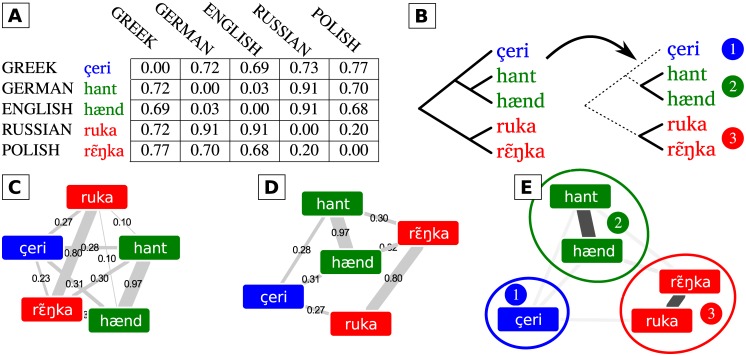
Workflows for automatic cognate detection. In LingPy, cognate detection is treated as a hierarchical clustering task. After distances or similarities between word pairs have been determined (A), a hierarchical clustering algorithm is applied to the matrix and terminates when a certain threshold is reached (B). Similarity networks start from a graph-representation of the similarity or distance matrix (C). In a first step, edges whose score exceeds a certain threshold are removed from the graph (D). In a second step, state-of-the-art algorithms for community detection are used to partition the graph into groups of cognate words (E).

A second method provided by LingPy, the Edit Distance approach, takes the normalized Levenshtein distance [46], between all word pairs in the same meaning slot and clusters these words into potential cognate sets using a flat version of the UPGMA algorithm [47] which terminates once a certain threshold of average distances between all words is reached. This general procedure of flat clustering, which is also employed for the two remaining cognate detection methods provided by LingPy, is illustrated in Fig 1A and 1B.

#### Cognate Detection using the Sound Class Algorithm

A third method available in the LingPy package, the SCA method, uses the same threshold-based clustering algorithm as the Edit Distance but employs distance scores derived from the Sound-Class Based Alignment (SCA) method [19]. This method for pairwise and multiple alignment analyses uses expanded sound class models along with detailed scoring functions as its basis. In contrast to previous alignment algorithms [48], the SCA algorithm takes prosodic aspects of the words into account and is also capable of aligning within morpheme boundaries, if morpheme information is available in the input data [19].

#### Cognate Detection using the LexStat method

The last publicly available method we tested, the LexStat method, is again based on flat UPGMA clustering, but in contrast to both the Edit-Distance method and the SCA method, it uses language-specific scoring schemes which are derived from a Monte-Carlo permutation of the data [19]. This permutation, by which the wordlists of all language pairs are shuffled in such a way that words denoting different meanings are aligned and scored, is used to derive a distribution of sound-correspondence frequencies under the assumption that both languages are not related. The permuted distribution is then compared with the attested distribution, and converted into a language-specific scoring scheme for all language pairs. Using this scoring scheme, the words in the data are aligned again, and distance scores are derived which are then used as the basis for the flat cluster algorithm.

#### Differences between algorithms

In order to illustrate the differences between these four algorithms, we analysed the test set by Kessler [49]. This dataset is particularly interesting for the task of cognate detection, since the sample of languages contains not only four Indo-European languages with different degrees of genetic affiliation, but also unrelated languages from different language families. When running the algorithm with default thresholds as proposed in List [19], LexStat performs best, showing the smallest amount of false positives and false negatives, followed by SCA, Edit-Distance, and Turchin. When looking at specific results of this analysis, like the cognate judgments for the concept ‘there’, given in Table 4, for example, we can immediately see the shortcomings of the language-independent methods. The Turchin method (T), for example, links Albanian [aty] and Navajo [ʔaːdi] as cognate, where these are a clear chance resemblance in the consonant class structure. Note that initial vowel is treated identical with initial glottal stop in the Turchin method, following the original sound class proposal by [45].

**Table 4:** Cognate detection algorithms in LingPy. Columns show the performance of cognate identification for the given wordforms in the International Phonetic Alphabet (IPA). The algorithms are the **T**urchin, **E**dit distance, **S**ound Class Algorithm, and **L**exStat methods. Italic numbers indicate false positives (forms incorrectly identified as cognate) and bold numbers indicate false negatives (forms incorrectly identified as not cognate) in comparison with the **G**old Standard.

Language	Word	IPA	T	E	S	L	G
Albanian	aty	aty	*1*	1	1	1	1
English	there	ðεr	**2**	**2**	**2**	2	2
French	là	la	3	*3*	3	3	3
German	da	daː	**4**	**4**	**4**	2	2
Hawaiian	laila	laila	5	*3*	5	4	4
Navajo	’áadi	ʔaːdi	*1*	5	6	5	5
Turkish	orada	ora	6	6	7	6	6

The Edit Distance (E) method also identifies a chance resemblance by proposing that French [la] and Hawaian [laila] are cognate. The Edit-Distance method is especially prone to identifying chance similarity as cognacy, and this risk increases as languages get more and more different [15]. The threshold of the SCA method (S) is too low to identify any cognate set for the concept ‘there’. Only the LexStat method (L) correctly identifies English [ðεr] and German [daː] as cognates, but not due to the phonetic similarity of the words, but due to the fact that matches of English [ð] and German [d] recur frequently in the dataset.

#### Similarity Networks

All the above cognate detection methods currently use a rather simple flat clustering procedure. The basis of this procedure is a clustering algorithm which terminates when average distances among sequences exceed a certain threshold. In evolutionary biology, the task of homolog detection is often approached from a *network perspective*. In *similarity networks*, for example, gene or protein sequences are modeled as the nodes of a network, and edges between the nodes are drawn with weights representing the pairwise similarities [50, 51]. Homolog detection is then modeled as a network partitioning task by which the network is divided into subgraphs with some objective criterion being used to define the best partition of the original network. While originally developed for the application in evolutionary biology, sequence similarity networks are now increasingly being tested on linguistics data [52, 53] and it was proposed that they might not only help to detect both genetically related words as well as words which have been borrowed [54]. Many strategies for network partitioning exist. The most common methods used in biology are Markov Clustering [55], *k*-means [56], and Affinity Propagation [57]. *k*-means has the strong disadvantage that it requires that the number of clusters into which the data shall be partitioned needs to be specified in advance. Tests in evolutionary biology have further shown that Markov Clustering outperforms Affinity Propagation [58]. This finding suggests that Markov Clustering would be an ideal choice for linguistic applications. However, when testing the approach on our training data, the results were inconclusive, and no real improvement compared with the default clustering algorithm used in LingPy could be observed.

For this study, we followed List et al. [53] in testing a partitioning approach which was originally developed for the task of community detection in social network analysis [59] and has shown to perform with a high accuracy: The Infomap algorithm [33] uses random walks to identify the best way to assign the nodes in a network to distinct communities. In order to convert the matrix of pairwise distances between words into a graph, we first define a threshold, and then add edges between all words whose pairwise distance is below the threshold. The edge weight is the distance score converted to a similarity score by subtracting it from 1. We use the pairwise distance matrices produced by the LexStat method, since this was shown to outperform the other three methods implemented in LingPy [19]. How cognate detection is modeled as a graph partitioning problem applied to similarity networks is displayed in more detail in Fig 1C and 1D.

#### Evaluation

It is not necessarily an easy task to compare how well an algorithm for automatic cognate detection performs in comparison with a “gold” standard. In our study, our gold standard are the expert cognate decisions by historical linguists using the comparative method. Scholars often use pairwise scores [32] for evaluation. In these scores, all words in a concept slot are assembled into pairs. The pair score is then calculated by comparing how many pairs in the gold standard are identically clustered by the algorithm, and vice versa. This is simple and straightforward, since, for pairs, there are only two possible decisions, namely whether they are cognate or not. We can then simply count how many pairs in the gold standard are also judged to be cognate by the algorithm, or how many pairs proposed to be cognate by the algorithm are also cognate according to the gold standard. The advantage of this score is that we can directly convert it into an intuitive notion of *false positives* and *false negatives* versus *true positives* and *true negatives*.

Breaking down the comparison of two different clusters into pairs is, however, problematic, since it has a strong bias in favoring datasets containing large amounts of non-cognate words [19]. In order to avoid these problems, we used B-Cubed scores as our primary evaluation method [37, 60, 61]. For the calculation of B-Cubed scores, we need to determine for each of the words the intersection of words between its cognate set in the gold standard and its cognate set proposed by the algorithm, as well as the size of the respective cognate sets. This is illustrated in Table 5 for a fictive test analysis of the five words in Fig 1, which wrongly clusters the Greek word with the English and the German word. For the B-Cubed precision we then average the size of the intersection divided by the size of the cognate set proposed by the algorithm for each of the words in our sample:

$$P=\frac{\frac{1}{3}+\frac{2}{3}+\frac{2}{3}+\frac{2}{2}+\frac{2}{2}}{5}=0.7\bar{3}$$ (1)

For the B-Cubed recall we average the intersection size divided by the cognate set size in the gold standard:

$$R=\frac{\frac{1}{1}+\frac{2}{2}+\frac{2}{2}+\frac{2}{2}+\frac{2}{2}}{5}=1.0$$ (2)

The B-Cubed F-Score is then computed as usual:

$$F=2\times \frac{P\times R}{P+R}=2\times \frac{0.7\bar{3}\times 1}{0.7\bar{3}+1}=0.\bar{846153}$$ (3)

**Table 5:** Preliminaries for B-Cubed score calculation. Cognate clusters, cluster size and cluster intersection for a fictive test analysis of the five words from Fig 1 compared to a gold standard.

Word	Cogn. Clusters		Cluster Size		Intersection
	Gold	Test	Gold	Test	
çeri	1	1	1	3	1
hant	2	1	2	3	2
hænd	2	1	2	3	2
ruka	3	2	2	2	2
r$\tilde{\varepsilon }$ŋka	3	2	2	2	2

### Threshold and Parameter Selection

**Fig 2:**
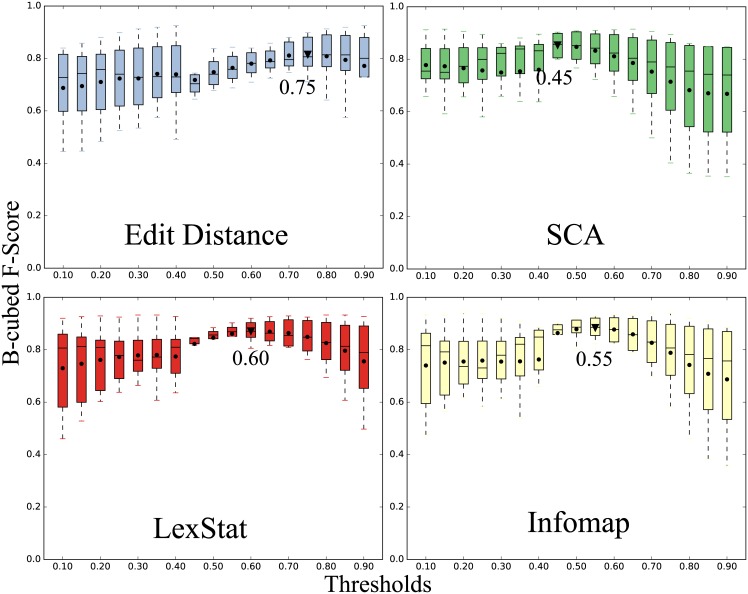
Determining the best thresholds for the methods. The *y*-axis shows the B-Cubed F-scores averaged over all training sets, and the *x* -axis shows the threshold for the 5 methods we tested. Infomap shows the best results on average, Edit Distance performs worst. Dots in the plots indicate the mean for each sample, with triangular symbols indicating the peak.

Apart from the Turchin method, all analyses require a threshold which ranges between 0 and 1, denoting the amount of similarity needed to judge two items as cognate. In order to find the most suitable threshold for each of the three methods, we used the expert cognate decisions in our training set and ran the analyses on these data with varying thresholds starting from 0.05 up to 0.95. Fig 2 shows box-plots of the training analyses for the four methods, depending on the threshold. As can be seen from this figure, all methods show a definite peak where they yield the best results for all datasets. In order to select the best threshold for each of the four methods, we selected the threshold which showed the best average B-Cubed F-Score (i.e. the best accuracy at recovering the known cognate sets). For the Edit Distance Method, the threshold was thus set to 0.75, for the SCA Method it was set to 0.45, for the LexStat Method, it was set to 0.60, and for the Infomap method, it was set to 0.55. The B-Cubed scores for these analyses are given in Table 6. These results indicate that the Infomap method performs best, followed by LexStat and SCA. Of the two worst-performing methods, the Turchin method performs worst in terms of F-Scores, but shows a much higher precision than the Edit-Distance method.

**Table 6:** Results of the training analysis to identify the best thresholds. Bold numbers indicate best values.

Method	Thr.	Prec.	Recall	F-Score
Turchin	-	0.8953	0.7276	0.8006
Edit Distance	0.75	0.8341	0.8101	0.8144
SCA	0.45	0.8650	0.8449	0.8529
LexStat	0.60	**0.9204**	0.8287	0.8700
Infomap	0.55	0.9012	**0.8712**	**0.8830**

## Results

We analyzed the datasets with each of the five methods described above, using the individual thresholds for each method, setting the number of permutations to 10,000, and using the default parameters in LingPy. For each analysis, we further calculated the B-Cubed scores to evaluate the performance of each method on each dataset.

Table 7 shows the averaged results of our experiments. While the LexStat method shows the highest precision, the Infomap method shows the highest recall and also the best general performance. The results are generally consistent with those reported by List [19] for the performance of Turchin, Edit Distance, SCA, and LexStat: The Turchin method is very conservative with a low amount of false positives as reflected by the high precision, but a very large amount of undetected cognate relations as reflected by the low recall. The Edit Distance method shows a much higher cognate detection rate, but at the cost of a high rate of false positives. The SCA method outperforms the Edit Distance, thus showing that refined distance scores can make a certain difference in automatic cognate detection.

**Table 7:** General results of the test analysis.

Method	Prec.	Recall	F-Score
Turchin	0.9108	0.7501	0.8175
Edit Distance	0.8397	0.8484	0.8396
SCA	0.8826	0.8492	0.8632
LexStat	**0.9227**	0.8488	0.8831
Infomap	0.9031	**0.8898**	**0.8942**

However, as the performance of LexStat and Infomap shows: Language-specific approaches for cognate detection clearly outperform language-independent approaches. The reason for this can be found in the specific similarity measure that is employed by the methods: the better performing methods are not based on surface similarities, but on similarities derived from previously inferred probability scores for sound correspondences. These methods are therefore much closer to the traditional comparative method than methods which employ simple surface similarities between sounds. Our experiment with the Infomap algorithm shows that a shift from simple agglomerative clustering approaches to a network perspective may further strengthen the results. Similarity networks have been successfully employed in evolutionary biology for some time now and should now become a fruitful topic of research in computational historical linguistics as well.

### Dataset Specific Results

**Fig 3:**
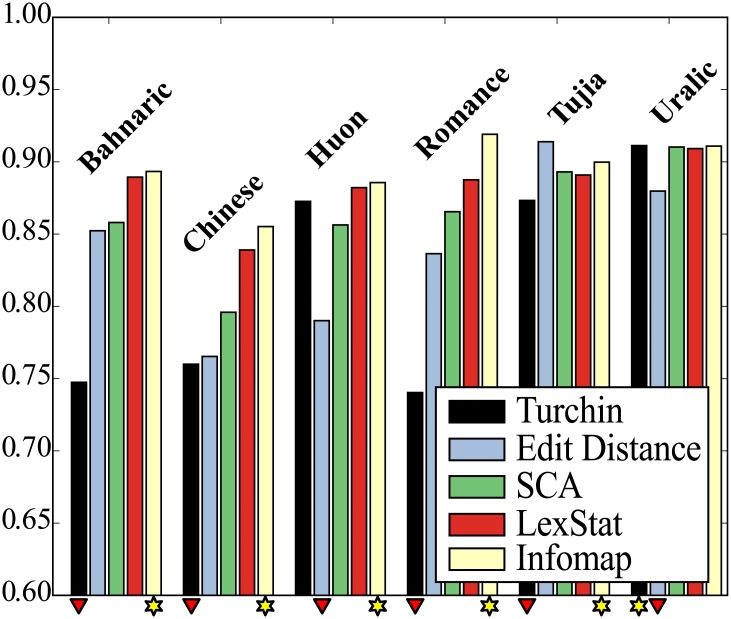
Individual test results (B-Cubed F-Scores). The figure shows the individual results of all algorithms based on B-Cubed F-Scores for each of the datasets. Results marked by a red triangle point to the worst result for a given subset, and results marked by a yellow star point to the best result. Apart from Uralic, our new Infomap approach always performs best, while the Turchin approach performs worst in four out of six tests.

**Fig 4:**
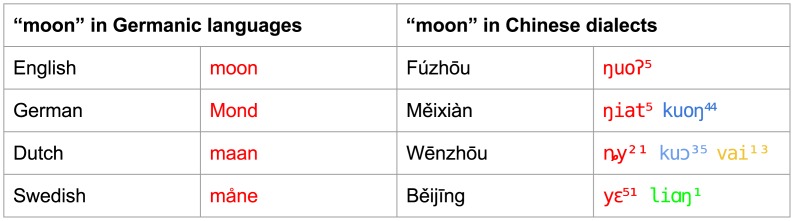
Partial and non-partial cognate relations. The word for “moon” in Germanic and Sinitic languages is mono-morphemic in Germanic languages, while it is usually compounded in Chinese dialects, with the first element in the compound meaning “moon” proper, while the second often originally meant “shine” or “glance”. The different cognate relations among the morphemes in the Chinese words make it impossible to give a binary assessment regarding the cognacy of the four words.

There are interesting differences between method performance across language datasets, with marked variation in cognate identification accuracy between different languages. Fig 3 shows the performance of the methods on the individual test sets, indicating which method performed best and which method performed worst. These results confirm the high accuracy of the LexStat method and the even better accuracy of the Infomap approach. All methods apart from the Turchin method perform the worst on the Chinese data. Since compounding is very frequent in Chinese, it is difficult to clearly decide which words to assign to the same cognate set. Often, words show some overlap of cognate material without being entirely cognate. This is illustrated in Fig 4, where cognates and partial cognates for Germanic and Sinitic languages are compared. We followed a strict procedure by which only words in which all morphemes are cognate are labelled as cognate [62], rather than loosely placing all words sharing a single cognate morpheme in the same cognate set [63]. Since neither of the algorithms we tested is specifically sensitive for partial cognate relations (for a recent proposal for this task, see [53]), they all show a very low precision, because they tend to classify only partially related words as fully cognate.

**Fig 5:**
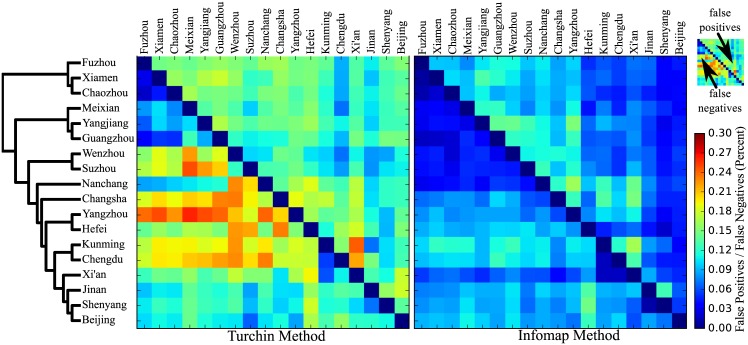
Distribution of true and false positives and true and false negatives.

The Turchin method has three extreme outliers in which it lags far behind the other methods: Chinese, Bahnaric and Romance. There are two major reasons for this. First, the Turchin method only compares the first two consonants and will be seriously affected by the problem of partial cognates discussed above. These partial cognates are especially prevalent in Chinese and Bahnaric where compounding is an important linguistic process. Second, a specific weakness of the Turchin method is the lack of an alignment and words are not exhaustively compared for structural similarities but simply mapped in their first two initial consonants. When there is substantial sound change, as is evident in both Bahnaric and some branches of Romance, this may lead to an increased amount of false negatives. Since the Turchin method only distinguishes 10 different sound classes and only compares the first two consonant classes in each word in the data, it is very likely to miss obvious cognates. The main problem here is that the method does not allow for any transition probabilities between sound classes, but treats them as discrete units. As a result, it is likely that the Turchin method often misses valid cognate relations which are easily picked up by the other methods. This shortcoming of the Turchin approach is illustrated in Fig 5, where the amount of true positives and negatives is contrasted with the amount of false positives and negatives in each dataset and for each of the five methods. This figure indicates that the Turchin method shows exceptionally high amounts of false negatives in Bahnaric and Romance. The clear advantage of the Turchin method is its speed, as it can be computed in linear time. Its clear disadvantage is its simplicity which may under certain circumstances lead to a high amount of false negatives.

The Edit-Distance method also performs very poorly. While, on average, it performs better than the Turchin approach, it performs considerably worse on the Chinese and Huon test sets. The reason for this poor performance can be found in a high amount of false positives as shown in Fig 5. While the Turchin method suffers from not finding valid cognates, the Edit-Distance method suffers from the opposite problem—identifying high amounts of false cognates. Since false positives are more deleterious for language comparison, as they might lead to false conclusions about genetic relationship [15], the Edit-Distance method should be used with very great care. Given that the SCA method performs better while being similarly fast, there is no particular need to use the Edit-Distance method at all.

**Fig 6:**
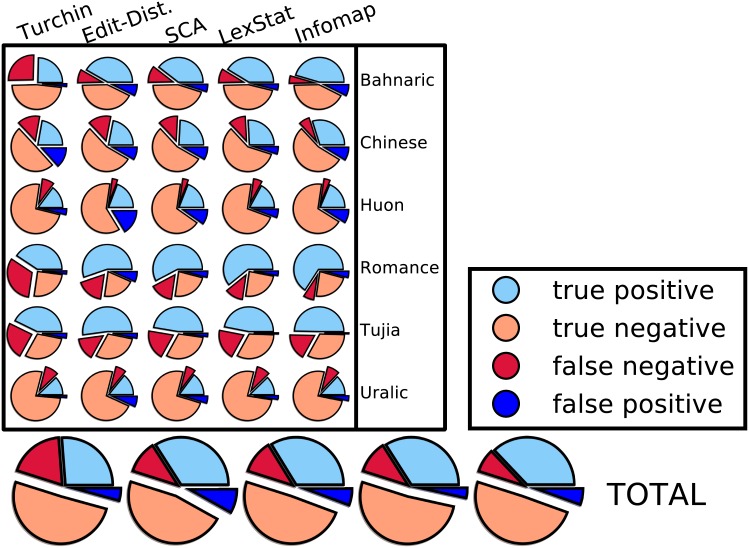
Comparing false positives and false negatives in the Chinese data. The figure compares the amount of false positives and false negatives, as measured in pairwise scores for the Turchin method and our Infomap approach for all pairs of language varieties in the Chinese test set. The upper triangle of the heatmaps shows the amount of false positives, while the lower triangle shows the amount of false negatives.

In Fig 6, we further illustrate the difference between the worst and the best approaches in our study by comparing false positives and false negatives in Turchin and Infomap across all language pairs in the Chinese data. As can be seen from Fig 5, the Turchin approach has about as many false positives as false negatives. The Infomap approach shows slightly more false positives than false negatives. This general picture, however, changes when looking at the detailed data plotted in Fig 6. Here, we can see that false positives in the Turchin approach occur in almost all dialect pairings, while the major number of cognates is missed in the mainland dialects (bottom of the *y*-axis). Infomap, on the other hand, shows drastically fewer false positives and false negatives, but while false negatives can be mostly observed in the Northern dialects (bottom of *y*-axis), false positives seem to center around the highly diverse Southern dialects (top of the *y*-axis). This reflects the internal diversity in Northern and Southern Chinese dialects, and the challenges resulting from it for automatic cognate detection. While word compounding is very frequent in the North of China, where almost all words are bisyllabic and bimorphemic, the Southern dialects often preserve monosyllabic words. While Northern dialects are rather homogeneous, showing similar sound systems and a rather large consonant inventories, Southern dialects have undergone many consonant mergers in their development, and are highly diverse. The unique threshold for cognate word detection overestimates similarities among the Southern dialects (upper triangle, left quarter), while it underestimates similarities among Northern dialects compared to Southern dialects (lower triangle, left quarter). What further contributes to this problem is also the limited size of the word lists in our sample, which make it difficult for the language-specific algorithms to acquire enough deep signal.

## Discussion

In this study we have applied four published methods and one new method for automated cognate detection to a set of six different test sets from five different language families. By training our data on an already published dataset of similar size, we identified the best thresholds to obtain a high accuracy for detecting truly related words for four out of the five methods (Edit-Distance: 0.75, SCA: 0.45, LexStat: 0.6, Infomap: 0.55). Using these thresholds, we tested the methods on our new gold standard, and found that most methods identified cognates with a considerable amount of accuracy ranging from 0.82 (Tuchin) to 0.89 (Infomap). Our new method, which builds on the LexStat method but employs the Infomap algorithm for community detection to partition words into cognate sets, outperforms all other methods in almost all regards, slightly followed by the LexStat approach. Given that the LexStat method and our Infomap approach are based on *language-specific* language comparison, searching for similar patterns in individual language pairs, our results confirm the superiority of cognate detection approaches which are closer to the theoretical foundation of the classical comparative method in historical linguistics. The Consonant Class Matching method by Turchin et al. confirmed worst in our experiment, followed by the Edit-Distance approach, which was criticized in earlier work [15]. While the major drawback of the Turchin approach is a rather large amount of false negatives, the Edit-Distance approach shows the highest amount of false positives in our test.

The method of choice may well depend on the task to which cognate detection is to be applied. If the task is to simply identify some potential cognates for future inspection and annotation, then a fast algorithm like the one by Turchin et al. should provide enough help to get started. This practice, which is already applied by some scholars [64], is further justified by the rather small amount of false positives. While the use of the Turchin method may be justified in computer-assisted workflows, the use of the Edit-Distance approach should be discouraged, since it lacks the speed advantages and is very prone to false positives.

When searching for deeper signals in larger datasets, however, we recommend using the more advanced methods, like SCA, LexStat or our new Infomap approach. LexStat and Infomap have the great advantage of taking regular sound correspondences into account. As a result, these methods tend to refuse chance resemblances and borrowings. Their drawback is the number of words needed to carry out the analysis. As we know from earlier tests [65], language-specific methods require at least 200 words for moderately closely related languages. When applied to datasets with higher diversity among the languages, the number of words should be even higher. Thus, when searching for cognates in very short word lists, we recommend using the SCA method to achieve the greatest accuracy. However, as demonstrated by the poorer performance of all methods on the Chinese language data where compounding has played a major role in word formation, language family specific considerations about the methods and processes need to be taken into consideration.

Our results show that the performance of computer-assisted automatic cognate detection methods has advanced substantially, both with respect to the applicability of the methods and the accuracy of the results. Moreover, given that the simple change we made from agglomerative to network-based clustering could further increase the accuracy of the results, shows that we have still not exhausted the full potential of cognate detection methods. Future algorithms may bring us even closer to expert’s judgments, and it seems worthwhile to invest time to increase the performance of our algorithms. Essential tasks for the future include (a) the work on parameter-free methods which do not require user-defined thresholds and state the results as probabilities rather as binary decisions, (b) the further development of methods for *partial cognate detection* [53], (c) approaches that search for cognates not only in the same meaning slot but across different meanings [66], and (d) approaches that integrate expert annotations to allow for a true iterative workflow for computer-assisted language comparison. A key problem to solve is the performance of these methods on larger datasets that trace language relationships to a greater depth. Most of our test cases in this paper are shallow families or subgroups of larger families. Deeper relationships between languages spoken in more complicated language situations are where the real challenge lies.

Currently automatic cognate detection algorithms are highly accurate at detecting a substantial proportion of the cognates in a lexical dataset. Tools like LingPy are already at a stage where they can act as a computer-assisted framework for language comparison. These tools therefore provide a powerful way of supplementing the historical linguistics toolkit by enabling linguists to rapidly identify the cognate sets which can then be checked, corrected, and augmented as necessary by experts. In regions where there has been an absence of detailed historical comparative work, these automated cognate assignments can provide a way to pre-process linguistic data from less well studied languages and speed up the process by which experts apply the comparative method. Additionally, these tools can be employed for exploratory data analysis of larger datasets, or to arrive at preliminary classifications for language families which have not yet been studied with help of the classical methods.

## References

[1] Greenhill SJ, Blust R, Gray RD. The Austronesian Basic Vocabulary Database: From bioinformatics to lexomics. Evolutionary Bioinformatics. 2008;4:271–283. 10.4137/ebo.s893 PMC2614200 19204825

[2] Dunn M. Indo-European lexical cognacy database (IELex). Nijmegen: Max Planck Institute for Psycholinguistics; 2012 URL: http://ielex.mpi.nl/.

[3] Greenhill SJ. TransNewGuinea.org: An online database of New Guinea languages. PLoS ONE. 2015;10(10):e0141563 10.1371/journal.pone.0141563 26506615 PMC4623503

[4] Kitchen A, Ehret C, Assefa S, Mulligan CJ. Bayesian phylogenetic analysis of Semitic languages identifies an Early Bronze Age origin of Semitic in the Near East. Proc Biol Sci. 2009 8;276(1668):2703–2710. 10.1098/rspb.2009.0408 19403539 PMC2839953

[5] Bowern C. Chirila: Contemporary and historical resources for the indigenous languages of Australia. Language Documentation and Conservation. 2016;10:1–44. Available from: http://nflrc.hawaii.edu/ldc/?p=1002.

[6] Fox A. Linguistic reconstruction. Oxford: Oxford University Press; 1995.

[7] Hammarström H, Forkel R, Haspelmath M, Bank S. Glottolog. Leipzig: Max Planck Institute for Evolutionary Anthropology; 2015 URL: http://glottolog.org.

[8] McMahon A, McMahon R. Language classification by numbers. Oxford: Oxford University Press; 2005.

[9] Embleton S. Lexicostatistics/glottochronology. From Swadesh to Sankoff to Starostin to future horizons In: Renfrew C, McMahon A, Trask L, editors. Time depth in historical linguistics. Cambridge: The McDonald Institute for Archaeological Research; 2000 p. 143–165.

[10] Holm HJ. The new arboretum of Indo-European “trees”. Journal of Quantitative Linguistics. 2007;14(2–3):167–214. 10.1080/09296170701378916

[11] Holman EW, Wichmann S, Brown CH, Velupillai V, Müller A, Bakker D. Explorations in automated lexicostatistics. Folia Linguistica. 2008;20(3):116–121.

[12] Wheeler WC, Whiteley PM. Historical linguistics as a sequence optimization problem: the evolution and biogeography of Uto-Aztecan languages. Cladistics. 2015;31:113–125. 10.1111/cla.12078 34758582

[13] Jäger G. Support for linguistic macrofamilies from weighted alignment. PNAS. 2015;112(41):12752–12757. 10.1073/pnas.1500331112 26403857 PMC4611657

[14] Campbell L. Comment on: Automated dating of the world’s language families based on lexical similarity. Current Anthropology. 2011;52:866–867.

[15] Greenhill SJ. Levenshtein distances fail to identify language relationships accurately. Computational Linguistics. 2011;37(4):689–698. 10.1162/COLI_a_00073

[16] Sidwell P. Comment on: Automated Dating of the World’s Language Families Based on Lexical Similarity. Current Anthropology. 2011;52:869–870.

[17] Trask RL. The dictionary of historical and comparative linguistics. Edinburgh: Edinburgh University Press; 2000.

[18] Ross MD, Durie M. Introduction In: Durie M, editor. The comparative method reviewed. New York: Oxford University Press; 1996 p. 3–38.

[19] List JM. Sequence comparison in historical linguistics. Düsseldorf: Düsseldorf University Press; 2014.

[20] Sidwell P. Austroasiatic dataset for phylogenetic analysis: 2015 version. Mon-Khmer Studies (Notes, Reviews, Data-Papers). 2015;44:lxviii–ccclvii.

[21] Saenko M. Annotated Swadesh wordlists for the Romance group (Indo-European family) In: Starostin GS, editor. The Global Lexicostatistical Database. RGU; 2015 Http://starling.rinet.ru/new100/tuj.xls.

[22] McElhanon KA. Preliminary Observations on Huon Peninsula Languages. Oceanic Linguistics. 1967;6(1):1–45. 10.2307/3622923

[23] Starostin GS. Annotated Swadesh wordlists for the Tujia group In: Starostin GS, editor. The Global Lexicostatistical Database. Moscow: RGU; 2013 URL: http://starling.rinet.ru/new100/tuj.xls.

[24] Běijīng Dàxué. Hányǔ fāngyán cíhuì 漢語方言詞匯 [Chinese dialect vocabularies]. Beijing: Wénzì Gǎigé; 1964.

[25] Syrjänen K, Honkola T, Korhonen K, Lehtinen J, Vesakoski O, Wahlber N. Shedding more light on language classification using basic vocabularies and phylogenetic methods. Diachronica. 2013;30(3):323–352. 10.1075/dia.30.3.02syr

[26] List JM, Cysouw M, Forkel R. Concepticon: A resource for the linking of concept lists. Leipzig: Max Planck Institute for Evolutionary Anthropology; 2016 URL: http://concepticon.clld.org.

[27] Wang F. Comparison of languages in contact The distillation method and the case of Bai. Taipei: Institute of Linguistics Academia Sinica; 2006.

[28] Hóu J. Xiàndài Hànyǔ fāngyán yīnkù 現代漢語方言音庫 [Phonological database of Chinese dialects]. Shànghǎi: Shànghǎi Jiàoyù; 2004.

[29] Hattori S. Japanese dialects In: Hoenigswald HM, Langacre RH, editors. Diachronic, areal and typological linguistics. The Hague and Paris: Mouton; 1973 p. 368–400.

[30] Zhivlov M. Annotated Swadesh wordlists for the Ob-Ugrian group (Uralic family) In: Starostin GS, editor. The Global Lexicostatistical Database. RGU; 2011 URL: http://starling.rinet.ru/new100/oug.xls.

[31] Beinborn L, Zesch T, Gurevych I. Cognate production using Character-based Machine Translation. In: Mitkov R, Park JC, editors. Proceedings of the Sixth International NLP Conference; 2013. p. 883–891.

[32] Bouchard-Côté A, Hall D, Griffiths TL, Klein D. Automated reconstruction of ancient languages using probabilistic models of sound change. PNAS. 2013;110(11):4224–4229. 10.1073/pnas.1204678110 23401532 PMC3600485

[33] Rosvall M, Bergstrom CT. Maps of random walks on complex networks reveal community structure. PNAS. 2008;105(4):1118–1123. 10.1073/pnas.0706851105 18216267 PMC2234100

[34] Mackay W, Kondrak G. Computing word similarity and identifying cognates with pair hidden markov models. In: Proceedings of the Ninth Conference on Computational Natural Language Learning; 2005. p. 40–47.

[35] Bergsma S, Kondrak G. Multilingual cognate identification using integer linear programming. In: Proceedings of the RANLP Workshop; 2007. p. 656–663.

[36] Berg-Kirkpatrick T, Klein D. Simple effective decipherment via combinatorial optimization. In: Proceedings of the 2011 Conference on Empirical Methods in Natural Language Processing; 2011. p. 313–321.

[37] Hauer B, Kondrak G. Clustering semantically equivalent words into cognate sets in multilingual lists. In: Proceedings of the 5th International Joint NLP conference; 2011. p. 865–873.

[38] Steiner L, Stadler PF, Cysouw M. A pipeline for computational historical linguistics. Language Dynamics and Change. 2011;1(1):89–127. 10.1163/221058211X570358

[39] Rama T, Kolachina P, Kolachina S. Two methods for automatic identification of cognates. In: Wielfaert T, Heylen K, Speelman D, editors. Proceedings of the 5th QITL Conference; 2013. p. 76–80.

[40] Ciobanu AM, Dinu LP. Automatic detection of cognates using orthographic alignment. In: Proceedings of the 52nd Annual Meeting of the ACL (Short Papers); 2013. p. 99–105.

[41] Jäger G, Sofroniev P. Automatic cognate classification with a Support Vector Machine. In: Proceedings of the 13th Conference on Natural Language Processing; 2016. p. 128–133.

[42] List JM, Moran S. An open source toolkit for quantitative historical linguistics. In: Proceedings of the ACL 2013 System Demonstrations. Stroudsburg: Association for Computational Linguistics; 2013. p. 13–18.

[43] Csárdi G, Nepusz T. The igraph software package for complex network research. InterJournal Complex Systems. 2006;p. 1695.

[44] Turchin P, Peiros I, Gell-Mann M. Analyzing genetic connections between languages by matching consonant classes. Journal of Language Relationship. 2010;3:117–126.

[45] Dolgopolsky AB. Gipoteza drevnejšego rodstva jazykovych semej Severnoj Evrazii s verojatnostej točky zrenija [A probabilistic hypothesis concerning the oldest relationships among the language families of Northern Eurasia]. Voprosy Jazykoznanija [Linguistic Inquiries]. 1964;2:53–63.

[46] Levenshtein VI. Dvoičnye kody s ispravleniem vypadenij, vstavok i zameščenij simvolov [Binary codes with correction of deletions, insertions and replacements]. Doklady Akademij Nauk SSSR. 1965;163(4):845–848.

[47] Sokal RR, Michener CD. A statistical method for evaluating systematic relationships. University of Kansas Scientific Bulletin. 1958;28:1409–1438.

[48] Kondrak G. A new algorithm for the alignment of phonetic sequences. In: Proceedings of the 1st North American chapter of the ACL conference; 2000. p. 288–295.

[49] Kessler B. The significance of word lists. Stanford: CSLI Publications; 2001.

[50] Méheust R, Zelzion E, Bhattacharya D, Lopez P, Bapteste E. Protein networks identify novel symbiogenetic genes resulting from plastid endosymbiosis. PNAS 2016;In press. 10.1073/pnas.1517551113 PMC4822624 26976593

[51] Corel E, Lopez P, Méheust R, Bapteste E. Network-thinking: Graphs to analyze microbial complexity and evolution. Trends Microbiol. 2016;24(3):224–237. 10.1016/j.tim.2015.12.003 26774999 PMC4766943

[52] Lopez P, List JM, Bapteste E. A preliminary case for exploratory networks in biology and linguistics In: Fangerau H, Geisler H, Halling T, Martin W, editors. Classification and evolution in biology, linguistics and the history of science. Stuttgart: Franz Steiner Verlag; 2013 p. 181–196.

[53] List JM, Lopez P, Bapteste E. Using sequence similarity networks to identify partial cognates in multilingual wordlists. In: Proceedings of the Association of Computational Linguistics 2016 (Volume 2: Short Papers). Berlin: Association of Computational Linguistics; 2016. p. 599–605.

[54] List JM, Pathmanathan JS, Lopez P, Bapteste E. Unity and disunity in evolutionary sciences: process-based analogies open common research avenues for biology and linguistics. Biology Direct. 2016;11(39):1–17. 27544206 10.1186/s13062-016-0145-2 PMC4992195

[55] van Dongen SM. Graph clustering by flow simulation [PhD Thesis]. University of Utrecht; 2000.

[56] MacQueen J. Some methods for classification and analysis of multivariate observations. In: Proceedings of the Fifth Berkeley Symposium on Mathematical Statistics and Probability. vol. 1. Berkeley: University of California Press; 1967. p. 281–297.

[57] Frey BJ, Dueck D. Clustering by passing messages between data points. Science. 2007;315:972–976. Available from: www.psi.toronto.edu/affinitypropagation. 10.1126/science.1136800 17218491

[58] Vlasblom J, Wodak SJ. Markov clustering versus affinity propagation for the partitioning of protein interaction graphs. BMC Bioinformatics. 2009;10:99 10.1186/1471-2105-10-99 19331680 PMC2682798

[59] Girvan M, Newman ME. Community structure in social and biological networks. PNAS. 2002;99(12):7821–7826. 10.1073/pnas.122653799 12060727 PMC122977

[60] Bagga A, Baldwin B. Entity-based cross-document coreferencing using the vector space model. In: Proceedings of the 36th Annual Meeting of the ACL; 1998. p. 79–85.

[61] Amigó E, Gonzalo J, Artiles J, Verdejo F. A comparison of extrinsic clustering evaluation metrics based on formal constraints. Information Retrieval. 2009;12(4):461–486. 10.1007/s10791-008-9066-8

[62] Ben Hamed M, Wang F. Stuck in the forest: Trees, networks and Chinese dialects. Diachronica. 2006;23:29–60. 10.1075/dia.23.1.04ham

[63] Satterthwaite-Phillips D. Phylogenetic inference of the Tibeto-Burman languages [PhD Thesis]. Stanford University. Stanford; 2011.

[64] Starostin G, Krylov P. The Global Lexicostatistical Database. Compiling, clarifying, connecting basic vocabulary around the world: From free-form to tree-form. Moscow: RGGU; 2011 URL: http://starling.rinet.ru/new100/main.htm.

[65] List JM. Investigating the impact of sample size on cognate detection. Journal of Language Relationship. 2014;11:91–101.

[66] Wahle J. An approach to cross-concept cognacy identification. In: Bentz C, Jäger G, Yanovich I, editors. Proceedings of the Leiden Workshop on Capturing Phylogenetic Algorithms for Linguistics. Tübingen; 2016. Available from: 10.15496/publikation-10060.

